# Anti-Fatigue Activity of Corn Protein Hydrolysate Fermented by Lactic Acid Bacteria

**DOI:** 10.3390/nu17020199

**Published:** 2025-01-07

**Authors:** Nan Hu, Jingyi Sun, Yujia Cao, Hongji Zhao, Meng Sun, Guanlong Li, Xiaolan Liu, Shanzi Cong

**Affiliations:** Heilongjiang Provincial Key Laboratory of Corn Deep Processing Theory and Technology, College of Food and Bioengineering, Qiqihar University, Qiqihar 161006, China

**Keywords:** corn protein hydrolysate, anti-fatigue, fermentation, gut microbiota, lactic acid bacteria

## Abstract

Objectives: This study aimed to clarify the effect of lactic acid bacteria-fermented corn protein hydrolysate (FCH) on fatigue in mice and explore the connection between fatigue-related indicators and intestinal microbial flora. Methods: The fatigue model of mice was constructed by exercise endurance experiment. The anti-fatigue level of FCH was evaluated by measuring physiological and biochemical indexes in mouse serum, liver and skeletal muscle. The relationship between FCH, intestinal flora and fatigue was explored through the analysis of intestinal microbial diversity in mice, and the anti-fatigue mechanism of FCH was further analyzed. Results: The results showed that the weight-bearing swimming time of mice was prolonged by 1.96 times, and the running time of mice was prolonged by 2.63 times in the high-dose FCH (FCH-H) group. Moreover, the lactic acid contents in the blood were reduced by 16.00%, and lactate dehydrogenase activity and urea nitrogen contents basically returned to the normal level. Meanwhile, the malondialdehyde contents were reduced by 31.24%, and superoxide dismutase activity and glutathione contents were increased by 1.84 times and 1.72 times, respectively. In addition, the glycogen contents of the body were restored, and the muscle glycogen and liver glycogen were increased by 1.81 and 5.81 times, respectively. Analysis of intestinal microbial flora diversity in mice showed that the highest relative abundance was *Lactobacillus*, and the FCH group could recover and even increase its relative abundance. *Lactobacillus* was significantly positively correlated with muscle glycogen and SOD. Conclusions: FCH can alleviate fatigue by regulating fatigue-related indicators and improving the intestinal microbial flora of the organism.

## 1. Introduction

The accelerating pace of modern life results in increased pressure and fatigue has become a common problem. Fatigue is a sub-health state, mainly due to physical or mental labor caused by increased energy consumption of various organs, resulting in a temporary decline in working ability and physical function [1,2]. Individuals who feel tired often manifest reduced exercise ability and work efficiency, and long-term fatigue often increases the incidence of various diseases, such as multiple sclerosis, Parkinson’s disease, depression, etc., which seriously affects normal work and life [3,4].

At present, the methods for relieving fatigue mainly include sleep, tobacco, dietary supplements, etc. [5,6,7]. Dietary supplements can restore normal physical functions by ingesting nutrients needed by the body. According to reports, consuming foods rich in polysaccharides, proteins, and peptides can effectively alleviate fatigue [8]. Wang et al. reported that the polypeptides extracted from sea cucumbers have good anti-fatigue function [9]. The high doses of sea cucumber peptides can prolong the exhaustive swimming time and forelimb grip strength of mice. Qiao et al. showed that the polysaccharides extracted from *Ribes stenocarpum* Maxim have strong anti-fatigue activity and can significantly increase the liver glycogen and muscle glycogen contents of mice [10]. Lu et al. found that fish protein hydrolysates can prolong the swimming time of mice, reduce the accumulation of lactic acid in the blood, and have a certain anti-fatigue function [11]. The above results clearly show that food supplementation is an effective means to relieve fatigue. Searching for foods that are inexpensive, abundant, contain anti-fatigue functional ingredients, and capable of being processed for enhancing contents of functional ingredients will become the main development direction of functional foods in the future.

Corn gluten meal is a byproduct of corn starch processing, with a protein content of over 60% and high hydrophobicity. It is commonly used as a raw material for animal feed. Corn gluten meal is limited in its application in food due to its lack of essential lysine and tryptophan for the human body [12]. By modifying corn gluten meal, not only can the amino acid composition of corn gluten meal be reshaped, but its physiological functions can also be improved, such as antioxidant activity, blood pressure-lowering effects, liver protection, and promotion of alcohol metabolism [13,14]. However, the modified corn gluten meal (corn protein hydrolysate, CPH) has a higher content of bitter peptides, resulting in a poorer flavor of the CPH [15]. Reprocessing CPH through fermentation can effectively improve its unpleasant flavor and further reduce its molecular weight, endowing it with stronger functionality and antioxidant properties [16]. Cui et al. used *Bacillus*, *Lactobacillus*, and *Hansenula* strains to co-ferment soybeans and studied the nutritional properties and anti-fatigue ability of the products and found that the contents of polypeptides, total phenols, and total flavonoids were significantly increased after fermentation, and its antioxidant activity was enhanced [17]. Moreover, fermented soybean products could increase the contents of liver glycogen in mice, alleviate the liver damage caused by exercise, and show a good anti-fatigue function [18]. Fang et al. studied the effects of soy protein hydrolysate fermented by *Lactobacillus acidophilus* on the anti-fatigue of mice and showed that the weight-bearing swimming time of the experimental group was significantly increased, indicating that the fermented soy protein hydrolysate had an anti-fatigue function [19]. Zhang et al. used lactic acid bacteria to ferment wheat protein hydrolysate and showed that it could increase the contents of liver glycogen and muscle glycogen in mice, prolong the exhaustive swimming time, and had an anti-fatigue ability [20]. These results show that plant-derived polypeptides have a good effect on relieving fatigue. In addition, lactic acid bacteria fermentation is also a good modification method for plant-derived polypeptides. At present, there are few reports on the potential anti-fatigue properties of FCH.

Numerous studies have shown that peptides have a certain positive impact on the body’s anti-fatigue function. This study evaluated the anti-fatigue effect of FCH in mice. The anti-fatigue level of FCH was evaluated by measuring physiological and biochemical indexes in mouse serum, liver, and skeletal muscle. Finally, the relationship between FCH, intestinal flora, and fatigue was explored through the analysis of intestinal microbial diversity in mice, and the anti-fatigue mechanism of FCH was further analyzed. The objective of the current work is to provide a theoretical basis for the functional study of FCH.

## 2. Materials and Methods

### 2.1. Subsection

The preparation method of FCH was according to Cong et al. [21].

CPHs were prepared by enzymatic hydrolysis of corn protein meal by a two-enzyme method. The CPH was dissolved in deionized water (substrate concentration 20%, *w*/*v*) and sterilized (121 °C, 30 min). The fermentation conditions of FCH were as follows: the 3:1 ratio of Lactobacillus rhamnosus and Lactobacillus fermentosa for inoculation (4% with the viable count of lactic acid bacteria of about 10^6^ CFU/mL, *v*/*v*), CPH concentration of 20.50%, the addition of 5% fructose syrup (*v*/*v*), and the fermentation at 39 °C for 24 h.

### 2.2. Experimental Animal Design

Ninety-six healthy male ICRs (18–22 g, 4-week-old, Institute of Cancer Research) were purchased from Changchun Yisi Experimental Animal Research Center (Changchun, China) (permit number: SCXK (yue) 2023-0017). The animal experiment was approved on 21 March 2023 by the Animal Ethics Committee of the College of Food and Bioengineering, Qiqihar University (Approval No. 2023-005). The conditions of temperature and humidity were 25 ± 1 °C and 55 ± 5%, respectively. The mice were fed with free access to water and food.

The mice were randomly divided into 6 groups according to their body weight, with 16 mice per group. After adapting to the environment for one week, the mice swimming screening experiment was carried out, and the mice that could not swim or had uncoordinated swimming postures were excluded. Ten mice were selected in each group for the follow-up experiment. The 6 groups were the blank control group (BCG, normal saline), fatigue model group (FMG, normal saline), positive control group (CPH-M, 250 mg/kg⋅bw of CPH), low-dose group (FCH-L, 125 mg/kg⋅bw of FCH), medium-dose group (FCH-M, 250 mg/kg⋅bw of FCH), and high-dose group (FCH-H, 500 mg/kg⋅bw of FCH), of which the FMG group did not participate in the determination of exercise endurance. The experiment lasted 28 days, and the initial weight of the mice was recorded. Weight was weighed once a day before feeding. After intragastric administration and resting for 30 min, swimming training was performed. The first week of swimming training was 20 min, and after that, it was increased by 5 min per week.

### 2.3. Determination of Weight-Bearing Swimming Time and Running Time

After 30 min of intragastric administration on the 26th day, the tails of the mice were wound with a lead block (10% of body weight). Then, the mice were forced to swim in a water pool (25 ± 1 cm, 25 ± 1 °C). Weight-bearing swimming time was recorded when the head of the mice under the water was more than 8 s.

After 30 min of intragastric administration on the 27th day, the mice in each experimental group were placed on the rotating rod in turn, and the rotating speed was slowly adjusted to 15 r/min. The first three times were pre-experiments, and the timing starts from the fourth time. Running time was recorded when the mice fell from the rod due to muscle fatigue. If the mice did not fall from the rotating rod within 30 min, the running time was recorded as 30 min.

### 2.4. Determination of Anti-Fatigue Relevant Physiological and Biochemical Indexes in Mice

After 30 min of the last intragastric administration, the mice (excluding the BCG group) without load were forced to swim in a water pool (30 ± 1 °C) for 30 min. Then, the mice were removed and wiped dry. After resting for 20 min, blood samples were collected from the orbital sinuses of mice, and skeletal muscles and internal organs such as liver were obtained by autopsy. The organ index was calculated according to the following formula.

Organ index (%) = organ mass (g)/mouse body weight (g) × 100%

The blood samples were placed at 4 °C overnight, centrifuged at 3000× *g* for 10 min, and the supernatant was serum. The liver and skeletal muscle were prepared into 10% liver homogenate and skeletal muscle homogenate with pre-cooled normal saline, centrifuged at 3000× *g* for 10 min. The concentrations of serum urea nitrogen (BUN), serum lactate (LA), serum lactate dehydrogenase (LDH), liver superoxide dismutase (SOD), liver glutathione (GSH), liver malondialdehyde (MDA), liver glycogen (LG), and muscle glycogen (MG) were determined using an assay kit (Jianglai Biotechnology Co., Ltd., Shanghai, China).

### 2.5. Detection of Intestinal Microbial Flora in Mice

The cecum samples were collected, 2 g per mice. The cecum samples were frozen in liquid nitrogen and then sent to Meiji Biomedical Technology Co., Ltd. (Shanghai, China). To determine the microbial diversity of mice cecum bacteria contents, the sample DNA was extracted using the TruSeqTM DNA Sample Prep Kit. After confirming purity, the V3–V4 region of the 16 S rDNA gene was amplified from DNA samples using the primer pair 338 F/806 R (338 F: 5′-ACTCCTACGGGAGGCAGCAG-3′, 806 R: 5′-GGACTACHVGGGTWTCTAAT-3′). The Illumina MiSeq platform was selected for sequencing, and the analysis method was the same as Hu et al. [22].

### 2.6. Data Analysis

The above tests were repeated at least three times, and the test results were expressed as mean ± standard deviation. Origin 2022 software was used to analyze the data and draw charts. SPSS Statistics 20.0 (IBM, Armonk, NY, USA) was used for significance analysis. The heatmap was visualized by TBtools V 2.01 (2023). Principal coordinates analysis (PCoA), statistical analysis, and mapping were performed with R language (version 3.3.1). Statistical analysis of different groups was performed using one-way analysis of variance (ANOVA) and Dunnett’s multiple comparison tests. Statistical significance was set at * *p* < 0.05 or ** *p* < 0.01.

## 3. Results

### 3.1. Effects of FCH on the Body Weight Change and Organ Index

**Figure 1:**
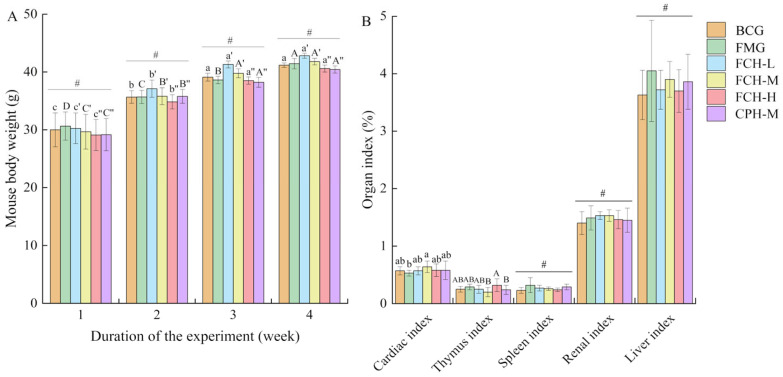
The effect of FCH on mice body weight and organ index. (**A**) The effect of FCH intervention on the body weight of mice. (**B**) The effect of FCH intervention on the organ index of mice. Data are shown as the mean ± SD (*n* = 6). Different large and lowercase letters in the figure indicate the significant difference between the groups (*p* < 0.05), and # indicates that there is no significant difference within the group (*p* > 0.05). BCG: blank control group; FMG: fatigue model group; CPH-M: medium dose group of CPH; FCH-L: low-dose group of FCH; FCH-M: medium-dose group of FCH; FCH-H: high-dose group of FCH.

Body weight change and organ index can reflect whether the administration of CPH and FCH has an impact on the health of mice. The weight of the six experimental groups of mice showed a steady upward trend during the test schedule (Figure 1A). During the 4 weeks of the experiment, the weight of the mice in each group increased by an average of 11.58 g. At the end of the experiment, the weight of the mice in each group reached 40.42–42.81 g. There were no significant differences between the experimental groups and the BCG group (*p* > 0.05), and the trend of weight gain was consistent. There were no significant differences between the spleen, kidney, and liver indexes of the mice in each group (*p* > 0.05) (Figure 1B). In the thymus index, the FCH-H group was significantly higher than the CPH-M group and the FCH-M group (*p* < 0.05), and no significant differences with other groups (*p* > 0.05) were observed. In the heart index, the FCH-M group was significantly higher than the FMG group (*p* < 0.05), and no significant differences with other groups (*p* > 0.05) were observed. In addition, no significant abnormalities and death were observed in the mice during the experiment. The above results show that each dose group of the FCH and the CPH had no apparent effect on the health of the mice.

### 3.2. Effect of FCH on Exercise Endurance

**Figure 2:**
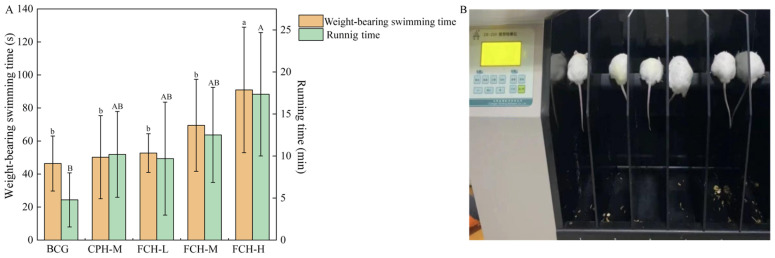
The effect of FCH on weight-bearing swimming and running time. (**A**) Mouse weight-bearing swimming and running time. (**B**) Mouse rotarod test process. Data are shown as the mean ± SD (*n* = 6). Different large and lowercase letters in the figure indicate significant differences between groups (*p* < 0.05). BCG: blank control group; CPH-M: medium-dose group of CPH; FCH-L: low-dose group of FCH; FCH-M: medium-dose group of FCH; FCH-H: high-dose group of FCH.

The test of weight-bearing swimming and running time in mice is one of the main indicators to study the anti-fatigue effect (Figure 2A). With increased FCH dose, the weight-bearing swimming time and running time of mice showed a steady upward trend. In the mouse weight-bearing swimming test, the final weight-bearing swimming time of the BCG group could reach 46.36 s after swimming training within the test schedule. The CPH-M group, the FCH-L group, and the FCH-M group showed no significant differences from the BCG group (*p* > 0.05). With further increase in the concentration of FCH, the weight-bearing swimming time of mice in the FCH-H group was significantly improved (*p* < 0.05), reaching 1.96 times that of the BCG group. The same results were obtained in the mouse rotarod test. The running time for the BCG group was 4.78 min. Compared with the BCG group, the running times for the CPH-M group, the FCH-L group, and the FCH-M group were slightly improved, but there were no significant differences (*p* > 0.05). The FCH-H group showed improved running time, reaching 3.63 times that of the BCG group (*p* < 0.05). It is concluded that FCH can effectively improve the weight-bearing swimming and running time of mice within a certain concentration range.

### 3.3. Effects of FCH on Anti-Fatigue Relevant Physiological and Biochemical Indexes in Mice

#### 3.3.1. Effect of FCH on Serum BUN, LA, and LDH Levels

**Figure 3:**
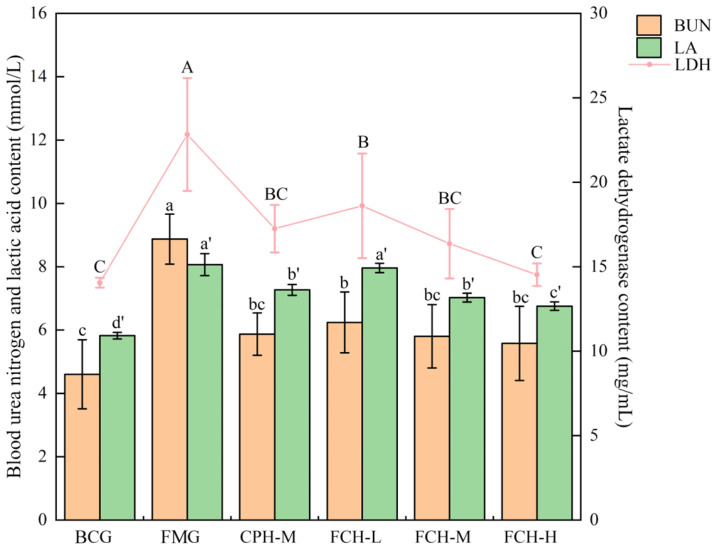
The effect of FCH on BUN, LA, and LDH. Data are shown as the mean ± SD (*n* = 6). Different large and lowercase letters in the figure indicate significant differences between groups (*p* < 0.05). BUN: urea nitrogen; LA: lactate; LDH: lactic dehydrogenase; BCG: blank control group; FMG: fatigue model group; CPH-M: medium dose group of CPH; FCH-L: low-dose group of FCH; FCH-M: medium-dose group of FCH; FCH-H: high-dose group of FCH.

The contents of BUN, LA, and LDH in mice are shown in Figure 3. The BUN contents of the BCG group were 4.46 ± 1.09 mmol/L, and the FMG group was 1.98 times that of the BCG group (*p* < 0.05). The BUN contents of the CPH-M group and the FCH groups were between the BCG group and the FMG group, at the level of 5.39–6.09 mmol/L. There was no significant difference between the other groups and the BCG group except for the FCH-L group (*p* < 0.05). The LA contents showed a similar trend to the BUN contents. The LA content of the FMG group was 8.06 ± 0.35 mmol/L, and the BCG group was 72.21% of the FMG group (*p* < 0.05). Compared with the FMG group, with increased FCH concentration, the LA content decreased significantly (*p* < 0.05). The LA content of the FCH-H group was 6.77 ± 0.13 mmol/L, which was 84.00% of the FMG group. The LA content of the CPH-M group was similar to the FCH-M group (*p* > 0.05). The LDH activity of the FMG group was 22.83 ± 3.34 ng/mL, and the BCG group was 61.54% of the FMG group (*p* < 0.05). With increased FCH concentrations, the LDH activities of FCH groups decreased significantly (*p* < 0.05), which were 81.52%, 71.66%, and 63.60%, respectively. The LDH activity of the FCH-H group was close to the BCG group (*p* > 0.05), and the CPH-M and FCH-M groups was similar (*p* > 0.05). Thus, FCH and CPH can effectively reduce the levels of BUN, LA, and LDH in the mice within a certain concentration range.

#### 3.3.2. Effects of FCH on MG and LG

**Figure 4:**
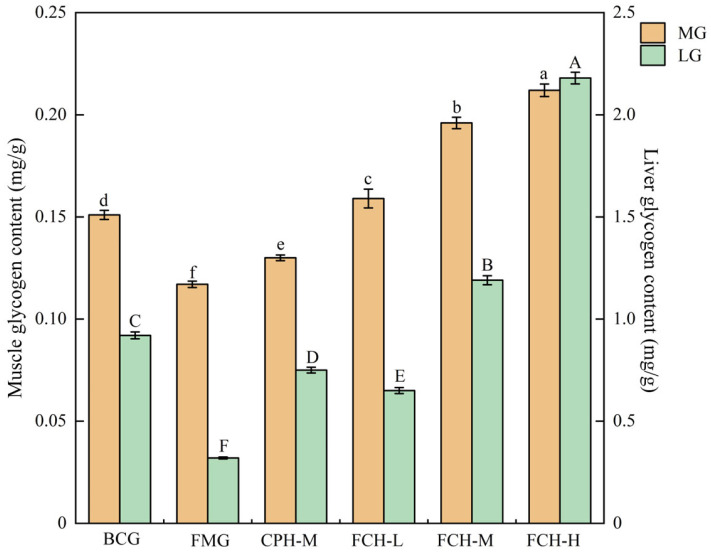
The effect of FCH on MG and LG. Data are shown as the mean ± SD (*n* = 6). Different large and lowercase letters in the figure indicate significant differences between groups (*p* < 0.05). MG: muscle glycogen; LG: liver glycogen; BCG: blank control group; FMG: fatigue model group; CPH-M: medium dose group of CPH; FCH-L: low-dose group of FCH; FCH-M: medium-dose group of FCH; FCH-H: high-dose group of FCH.

The contents of MG and LG in mice were shown in Figure 4. The MG content of the FMG group was 0.117 ± 0.002 mg/g, the BCG group was 1.29 times that of the FMG group (*p* < 0.05), and the MG content for the CPH-M group was between the FMG group and the BCG group (*p* < 0.05). With increased FCH concentrations, the MG contents of the FCH groups increased significantly, which were 1.36 times, 1.68 times, and 1.81 times that of the FMG group, respectively (*p* < 0.05). The LG content of the FMG group was 0.32 ± 0.005 mg/g, and the BCG group was 2.88 times that of the FMG group (*p* < 0.05). With increased FCH concentrations, the LG content of the FCH groups increased significantly, which was 2.03 times, 3.72 times, and 6.81 times that of the FMG group, respectively (*p* < 0.05). The LG contents of the CPH-M group was between the FMG group and the FMG-M group (*p* < 0.05). It is concluded that FCH and CPH can effectively increase the contents of MG and LG in mice within a certain concentration range, and the effect of FCH was significantly better than that of CPH.

#### 3.3.3. Effects of FCH on Liver SOD, MDA, and GSH

**Figure 5:**
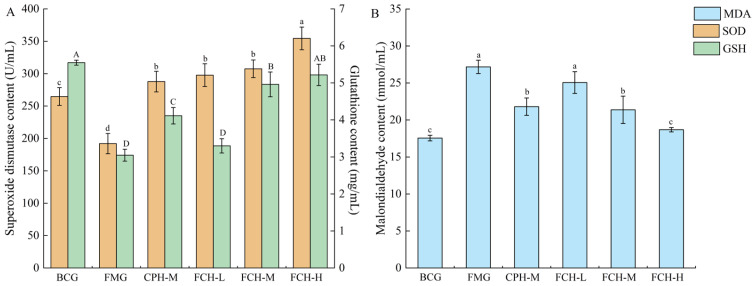
The effects of FCH on SOD, GSH, and MDA. (**A**) SOD and GSH contents in mouse liver. (**B**) MDA content in mouse liver. Data are shown as the mean ± SD (*n* = 6). Different large and lowercase letters in the figure indicate significant differences between groups (*p* < 0.05). MDA: Malondialdehyde; SOD: Superoxide dismutase; GSH: Glutathione; BCG: blank control group; FMG: fatigue model group; CPH-M: medium dose group of CPH; FCH-L: low-dose group of FCH; FCH-M: medium-dose group of FCH; FCH-H: high-dose group of FCH.

The SOD activity and GSH contents of mice were shown in Figure 5A. The SOD activity test revealed that the SOD activity of the FMG group was 192.02 ± 15.6 U/mL, and the BCG group was 1.38 times that of the FMG group (*p* < 0.05). With increased FCH concentrations, the SOD activities of FCH groups were significantly increased, which were 1.55 times, 1.60 times, and 1.84 times that of the FMG group, respectively (*p* < 0.05). The GSH content of the FMG group was 3.05 ± 0.16 mg/mL, and the BCG group was 1.82 times that of the FMG group (*p* < 0.05). Compared with the FMG group, the GSH content of the FCH-M group and the FCH-H group was increased by 1.63 times and 1.72 times, respectively (*p* < 0.05), and the FCH-H group was close to the BCG group (*p* > 0.05). The GSH content of the CPH-M group was at the intermediate level of the FCH-L group and the FCH-M group (*p* < 0.05).

The MDA content of mice was shown in Figure 5B. The MDA content of FMG group was 27.18 ± 0.9 mmol/mL, and the BCG group was 64.61% of the FMG group (*p* < 0.05). Compared with the FMG group, MDA contents of the FCH-M group and FCH-H group were significantly reduced, being 78.66% and 68.76% of the FMG group, respectively (*p* < 0.05), and the FCH-H group was similar to the BCG group (*p* > 0.05). These results indicate that FCH and CPH could effectively increase SOD activity, as well as the MDA and GSH contents of mice within a certain concentration range, and the effect of FCH on GSH contents was better than CPH.

### 3.4. Effects of FCH on Intestinal Microbial Flora

#### 3.4.1. Bacterial Alpha Diversity Analysis of Intestinal Microbial Flora in Mice

**Figure 6:**
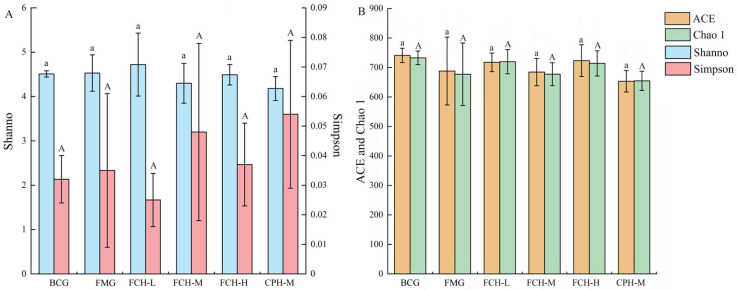
Alpha diversity index of mice intestinal microbial flora. (**A**) Shannon and Simpson index; (**B**) ACE and Chao 1 index. Data are shown as the mean ± SD (*n* = 6). Different large and lowercase letters in the figure indicate significant differences between groups (*p* < 0.05). BCG: blank control group; FMG: fatigue model group; CPH-M: medium dose group of CPH; FCH-L: low-dose group of FCH; FCH-M: medium-dose group of FCH; FCH-H: high-dose group of FCH.

The Shannon index and Simpson index were used to compare the species diversity of mice intestinal bacteria (Figure 6A). The Shannon index and Simpson index of the 6 groups ranged from 4.18 to 4.72 and 0.025 to 0.054, respectively, and there was no significant difference (*p* > 0.05). The ACE index and Chao 1 index were used to evaluate the species richness (Figure 6B). The ACE index of the 6 groups ranged from 653.26 to 740 and for the Chao 1 index ranged from 654.62 to 732, with no significant difference (*p* > 0.05). It can be concluded that FCH and CPH had no significant effect on changes in the bacterial diversity and abundance of mice intestinal microbial flora.

#### 3.4.2. Bacterial Beta Diversity Analysis of Intestinal Microbial Flora

**Figure 7:**
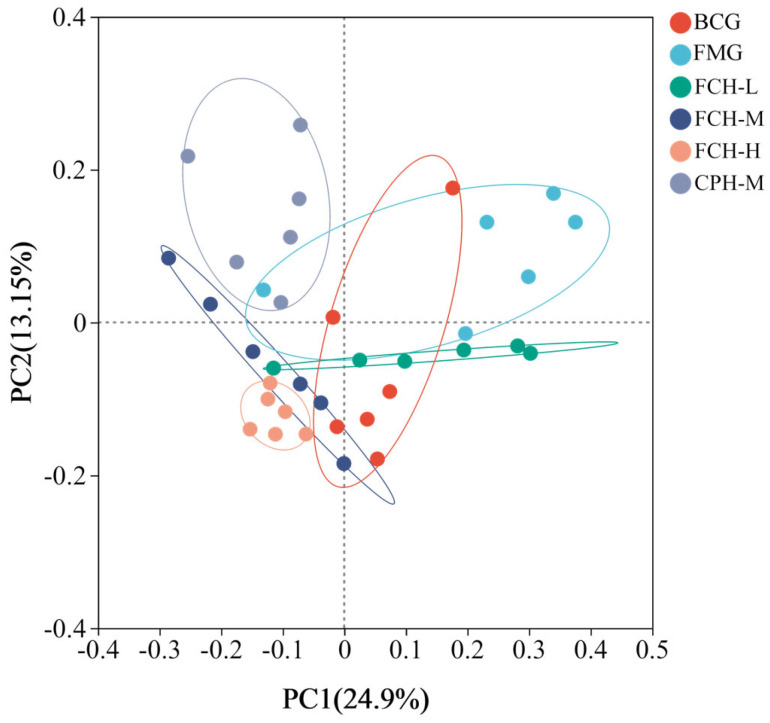
Beta diversity index of intestinal microbial flora. BCG: blank control group; FMG: fatigue model group; CPH-M: medium dose group of CPH; FCH-L: low-dose group of FCH; FCH-M: medium-dose group of FCH; FCH-H: high-dose group of FCH.

The β-diversity (intergroup diversity) of intestinal microbial flora in mice was analyzed by the PCoA method. The distance between the sample points represented the similarity of the microbial communities in the samples, and the closer the distance, the higher the similarity (Figure 7). Under the influence of PC1 and PC2, the six groups formed completely different taxonomic clusters. It is evident that exercise fatigue, FCH, and CPH had a greater impact on the structure of the intestinal microbial flora in mice. There was a certain intersection between the BCG group and the FMG group in Figure 7, indicating that there were certain individual differences in the microbial diversity of the intestinal microbial flora in mice. However, FCH groups had basically no intersection with the FMG group, and the FCH-L and FCH-M groups are more tended to than the BCG group. The FCH-H group had no intersection with the BCG group and the FMG group, but it was more tended to than the BCG group. The results showed that a certain concentration of FCH could effectively regulate the intestinal microbiota of mice to achieve a normal state. The intersection between the CPH group and the FMG group was very small, indicating that a certain concentration of CPH can also improve the poor state of intestinal microbiota caused by fatigue.

#### 3.4.3. Classification and Analysis of Intestinal Microbial Flora

**Figure 8:**
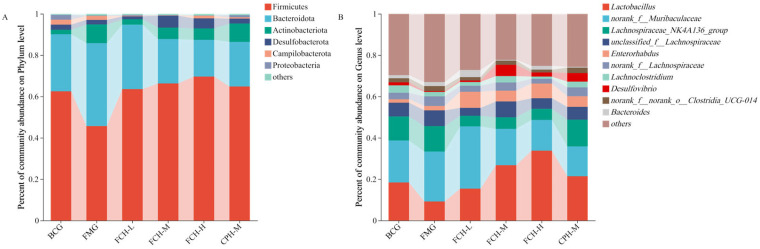
The phylum- (**A**) and genus- (**B**) level bacterial community structure of the intestinal microbial flora. BCG: blank control group; FMG: fatigue model group; CPH-M: medium dose group of CPH; FCH-L: low-dose group of FCH; FCH-M: medium-dose group of FCH; FCH-H: high-dose group of FCH.

Figure 8 shows the phylum- and genus-level bacterial community structure of the intestinal microbial flora in mice.

The relative abundance of Firmicutes was 62.49%, ranking first in phylum-level relative abundance (Figure 8A). The relative abundance of Firmicutes in the BCG group and the FMG group was 62.49% and 45.70%, respectively. The relative abundances of Firmicutes in the CPH-M group and the FCH groups were 64.82%, 63.58%, 66.31%, and 69.63%, respectively, which were higher than the BCG group and the FMG group. The relative abundance of Bacteroides was 40.11%, ranking second. The relative abundance of Bacteroides in the BCG group was 27.62% and in the FMG group was 40.11%. The relative abundance of Bacteroidetes in the CPH-M group and the FCH groups were 21.60%, 31.18%, 21.47%, and 17.75%, respectively, which were lower than the FMG group and closer to the BCG group. The third-highest relative abundance belonged to Actinomycetes, with a relative abundance of 9.06%. The relative abundance of Actinomycetes in the BCG group was 2.16%, while for the FMG group it was 9.06%. The relative abundances of Actinomycetes in the CPH-M group and the FCH groups were 8.90%, 2.60%, 5.55% and 5.57%, respectively, in between the FMG group and the BCG group. The relative abundance of the rest of the bacterial population was less than 5%, and the relative effect was small.

The relative abundance of *Lactobacillus* was the highest in genus-level relative abundance (Figure 8B). The BCG group was 18.41% and the FMG group was only 9.19%. The CPH-M group and the FCH groups were 21.42%, 15.42%, 26.77%, and 33.78%, respectively, which were higher than the BCG group and the FMG group. The second highest relative abundance was *Muribaculaceae*, and the relative abundance of the BCG group and the FMG group was similar, with 20.32% and 24.18%, respectively. The CPH-M group and the FCH groups were 14.43%, 30.12%, 17.57%, and 14.88%, respectively. The third-highest relative abundance belonged to the *Lachnospiraceae_NK4A136_group*, and the BCG and FMG groups was still similar, with 11.60% and 12.31%, respectively. The CPH-M group was 12.94%, which was close to the BCG group and the FMG group. The FCH groups were lower than the BCG group and the FMG group, with 5.13%, 5.60%, and 5.38%, respectively. The relative abundance of *unclassified_f_Lachnospiraceae* in the BCG group and the FMG group was similar, being 6.66% and 7.58%, respectively. The CPH-M group, the FCH-M group, and the FCH-H group were closer to the BCG group and the FMG group, with 6.20%, 7.65%, and 5.10%, respectively. The FCH-L group was 3.83%, which was slightly lower than other groups. The relative abundance of the remaining bacteria was less than 5%.

#### 3.4.4. Correlation Analysis Between Intestinal Microbial Flora and Fatigue-Related Biochemical Indexes

**Figure 9:**
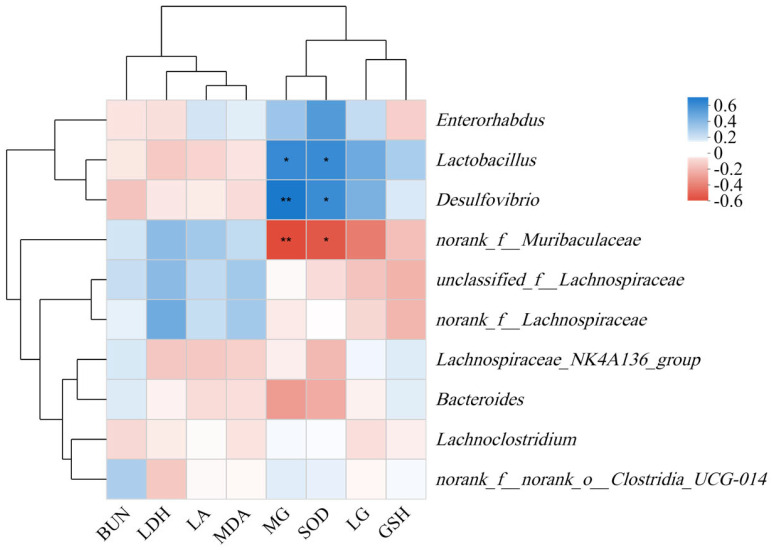
Correlation clustering heat map between intestinal microbial flora and fatigue indexes. * Indicates significant difference (*p* < 0.05), ** Indicates extremely significant difference (*p* < 0.01). BUN: urea nitrogen; LDH: lactate dehydrogenase; LA: lactate; MDA: malondialdehyde; MG: muscle glycogen; SOD: superoxide dismutase; LG: liver glycogen; GSH: glutathione.

The change in intestinal microbial flora in mice may be related to fatigue status. By analyzing the relationship between intestinal microbial flora and fatigue indexes in mice, the correlation between intestinal microbial flora and fatigue in mice was revealed (Figure 9). *Lactobacillus* was significantly positively correlated with MG and SOD, *Desulfovibrio* was significantly positively correlated with SOD (*p* < 0.05), and *Desulfovibrio* was highly significant positive correlated with MG (*p* < 0.01). However, *Muribaculaceae* was significantly negatively correlated with SOD (*p* < 0.05) and highly significantly negatively correlated with MG content (*p* < 0.01).

## 4. Discussion

The purpose of this study was to explore the anti-fatigue effect of FCH. The reduction in exercise endurance is the most intuitive manifestation of organism fatigue [23]. This study first tested the ability of mice to weight-bearing swimming and running. From the perspective of exercise performance, feeding FCH and CPH could significantly prolong the time of weight-bearing swimming and running of mice. Among them, the FCH-H group was extended by 44.58 s and 12.57 min, respectively. FCH and CPH were shown to have apparent anti-fatigue effects at a certain concentration. Fang et al. reported that mice fed with fermented soybean protein peptides showed an increase of 35.78% for the weight-bearing swimming time [19]. Zhang et al. reported the doubling of the running time of mice after 4 weeks of administration of the peptide isolated from *Hippocampus abdominalis* [24]. The above results are consistent with the findings of this study, showing that the peptides can relieve exercise fatigue within a certain concentration, and the effect of lower molecular weight peptides is more obvious. Compared with CPH, FCH contains large number of functional short peptides with lower molecular weight, which can participate in regulating sugar metabolism, reducing protein consumption in muscles, thereby reducing urea production and achieving an anti-fatigue effect [25,26].

When the organism does not acquire energy replenishment in time after intense exercise, proteins will decompose to form blood urea nitrogen, while it will also accelerate the glycolysis process to produce a large amount of LA, which increases the concentration of LA or H+ in the muscles and causes muscle fatigue [27,28]. This study found that feeding a certain concentration of FCH by gavage can reduce the levels of BUN and LA in mice, indicating that long-term intake of a certain concentration of FCH can effectively relieve organism fatigue. Wang et al. reported that gavage mice with mackerel peptides for 4 weeks reduced the levels of BUN and LA by 0.88 mmol/L and 180.86 ug/L, respectively [29]. Yin et al. showed that gavage mice with the Young Yum Pill for 3 weeks, the BUN and LA contents were reduced by 29% and 50%, respectively [30]. Compared with mackerel peptides and the Young Yum Pill, FCH can increase the glycogen reserve of mice and reduce the catabolism of proteins in mice, thereby achieving a better anti-fatigue effect [31]. The two forms of glycogen reserve in the organism are LG and MG, and the storage of energy determines the tolerance of exercise. When the organism has insufficient blood sugar during intense exercise, it needs to consume reserve glycogen to maintain blood sugar levels to meet the needs of the organism [32]. FCH can significantly increase the organism’s glycogen reserves. The contents of MG and LG in the FCH groups were significantly higher than the FMG group, and significantly higher than the BCG group at a certain concentration. Meanwhile, the increase in LG contents in mice was higher than that in the MG contents. This may be due to the fact that the organism preferentially consumes MG under the condition of stable blood sugar [33]. Therefore, the accumulation of LG in mice was higher than that of MG, similar to the results of Feng et al. and Lu et al. [33,34].

Strenuous exercise or muscle injury can cause LDH in skeletal muscle to penetrate into the blood, and LDH can catalyze the conversion of pyruvate to LA, reducing exercise tolerance [10]. This study showed that the FCH groups and the CPH group can significantly reduce the LDH activity in the blood, of which the FCH-H group has approached the level of the BCG group. Chen et al. showed that mice fed with spirulina peptides for 4 weeks had a 53.5% decrease in LDH activities [35]. Liu et al. reported that mice fed with peanut oligopeptides for 30 days had a 50% decrease in LDH activities [36]. By contrast, FCH can reduce the level of LDH in the blood more effectively. This may be related to the higher contents of branched-chain amino acids and phenylalanine in FCH [37]. It has been reported that branched-chain amino acids in CPH can promote the synthesis of skeletal muscle protein, repair skeletal muscle damage, and reduce LDH exudation, while hydrophobic amino acid residues such as Ala and Leu contained in CPH have the effect of scavenging free radicals and preventing lipid oxidation [38]. After strenuous exercise, the organism produces large number of free radicals, triggers lipid peroxidation, increases MDA contents, changes the permeability of cell membranes and, thus, leading to fatigue. In this study, FCH can effectively reduce MDA contents, and there is no significant difference between the FCH-H group and the BCG group. Zhong et al. showed that mice fed with soft-shelled turtle peptides for 30 days resulted in a 40.4% decrease in MDA contents [39]. Ye et al. reported that mice fed with sea cucumber peptides for 4 weeks had a 50% reduction in MDA [40]. The effect of FCH on MDA content reduction is better than that of soft-shelled turtle peptides and sea cucumber peptides. This may be due to the stronger antioxidant activity of CPH contributed by lactic acid bacteria fermentation, which accelerates the scavenging of superoxide free radicals in the organism, antagonizes the reactive oxygen clusters continuously generated by skeletal muscles during strong contraction, and reduces the loss of superoxide dismutase or due to the increase in SOD enzyme activity and GSH contents, resulting in the increase in the organism’s antioxidant activity and reducing the free radicals produced by strenuous exercise [41,42,43,44,45]. The results reported in this study are consistent with the previous findings cited above.

The intestinal microbial flora of mice was further measured to explore the relationship between FCH and fatigue in mice. Although there was no significant difference in the alpha diversity of the six groups (*p* > 0.05). However, the beta diversity showed that FCH and CPH could effectively regulate the intestinal microbiota of mice to keep in a normal state (Figure 7). Among the intestinal microbial flora of mice, the relative abundances of Firmicutes (45.70–69.63%) and Bacteroides (17.75–40.11%) were the highest. The abundance of Firmicutes in the FMG group was lower than the BCG group, and the abundance of Bacteroides was higher than the BCG group. By feeding a certain concentration of FCH, the relative abundance of Firmicutes in the intestinal tract of mice increased significantly, and the relative abundance of Bacteroidetes gradually decreased. Firmicutes mainly include a variety of beneficial bacteria, while Bacteroidetes are closely related to metabolic diseases in the organism [46]. In this study, the ratio of Firmicutes to Bacteroidetes in the FCH-H group was closer to the BCG group. It is concluded that FCH can achieve the purpose of anti-fatigue by regulating the homeostasis of the level of intestinal flora in mice. At the genus level, the relative abundance of *Lactobacillus* (9.19–33.78%) was the highest. The abundance of *Lactobacillus* in the BCG group was significantly higher than the FMG group. By feeding FCH, the relative abundance of *Lactobacillus* in the intestines of mice was significantly increased, the FCH groups were higher than the FMG group. Fang et al. also obtained similar results in the fatigue intervention experiment of fermented soy protein peptide in mice. Fermented products of *Lactobacillus* can improve the intestinal flora, increase the abundance of probiotics in the intestines of tired mice, and, thus, affect system metabolism by changing the host metabolome, so as to improve the organism’s metabolic capacity and anti-fatigue ability [17,19,47,48]. The increase in *Lactobacillus* contents in the intestines can significantly increase the contents of short fatty acids, activate peroxisome proliferators and receptor gamma coactivator 1α (PGC-1α), increase the production of ATP, provide energy for exercise, and improve exercise endurance performance [48]. In the correlation analysis, it was also found that *Lactobacillus* was significantly positively correlated with MG. It has been reported that *Lactobacillus* can affect the composition of intestinal microorganisms, regulate the genes related to glycogen synthesis in tissues (GSK-3β and Akt), and, thereby, affect the glycogen contents of the organism [49].

In conclusion, long-term intake of a certain concentration of FCH can effectively relieve organism fatigue. FCH can play an anti-fatigue effect by regulating the intestinal microbial flora of mice, and the anti-fatigue effect of FCH is better than that of CPH.

## 5. Conclusions

Long-term ingestion of a certain amount of peptide can effectively relieve fatigue caused by exercise. However, due to the different molecular weights of various peptides, there are also obvious differences in the effects on fatigue. This study aimed to reveal the effect of FCH on organism fatigue and explore the relationship between intestinal microorganisms and fatigue-related indicators. From the perspective of the exercise state of mice, ingesting FCH for 4 weeks could effectively improve the exercise endurance of mice as confirmed in the weight-bearing swimming and rotarod test of mice. In the determination of biochemical indicators, it was found that FCH could effectively reduce the contents of LA and LDH in the blood and restore the content of BUN to normal levels. Moreover, the activities of SOD and GSH were increased to varying degrees, and the contents of MDA were reduced significantly, while the glycogen reserve of the organism was restored. MG and LG were increased to varying degrees, and most experimental groups had exceeded the level of the BCG group. The results showed that both FCH and CPH had certain anti-fatigue effects. For the same dose, the effect of FCH was better than CPH. In the analysis of intestinal microorganisms in mice, *Lactobacillus* was the most abundant relative bacteria, followed by *norank_f_Muribaculaceae*. Different concentrations of FCH could restore or even increase the relative abundance of *Lactobacillus* and reduce the abundance of *norank_f_Muribaculaceae*. In the association analysis, *Lactobacillus* was significantly positively correlated with MG and SOD, while *norank_f_Muribaculaceae* was significantly negatively correlated with SOD and was significantly negatively correlated with MG. Fatigue of the organism may be related to the ratio of *Lactobacillus* and *norank_f_Muribaculaceae*, but the specific mechanism involved needs to be revealed by further experiments.

## Data Availability

Data is contained within the article.

## Abbreviations

The following abbreviations are used in this manuscript:

FCH	Lactic acid bacteria-fermented corn protein hydrolysate
CPH	Corn protein hydrolysates
BCG	Blank control group
FMG	Fatigue model group
CPH-M	Medium dose group of CPH
FCH-L	Low-dose group of FCH
FCH-M	Medium dose group of FCH
FCH-H	High dose group of FCH
BUN	Urea nitrogen
LA	Lactate
LDH	Lactic dehydrogenase
MG	Muscle glycogen
LG	Liver glycogen
MDA	Malondialdehyde
SOD	Superoxide dismutase
GSH	Glutathione
PCoA	Principal coordinates analysis
ACE	Abundance-based coverage estimator
ICR	Institute of cancer research
